# Secondary Aortic Dissection after Endoluminal Treatment of an Intramural Hematoma of the Thoracoabdominal Aorta: Endovascular Extension with Two Stent Grafts and Scarce Distal Landing

**DOI:** 10.1155/2013/714914

**Published:** 2013-12-03

**Authors:** Peter I. Kalmar, Peter Oberwalder, Peter Schedlbauer, Jürgen Steiner, Rupert H. Portugaller

**Affiliations:** ^1^Department of Radiology, Medical University of Graz, Auenbruggerplatz 9a, 8036 Graz, Austria; ^2^Department of Surgery, Medical University of Graz, Auenbruggerplatz 9a, 8036 Graz, Austria

## Abstract

Secondary dissection in the descending aorta after endovascular therapy may demand subsequent interventional procedures. This can set a particularly significant challenge for the endovascular specialist. When implanting an aortic prosthesis, a sufficient contact between the covered segment and the healthy vessel wall is advisable. However, our case shows that, in individual cases, it is indeed efficient to place an aortic stent graft on top of the distal end of the dissection. This is proven by a three-year follow-up CT-angiography.

## 1. Introduction

The management of dissections of the thoracoabdominal aorta with a marginal distal landing zone remains controversial. Surgical reconstruction provides a safe way to avoid the risk of visceral branch occlusion. In some cases, however, endovascular therapy should be considered when a scarce distal landing is possible. Herein, we report a case in which a thoracic aortic stent graft was distally extended in order to successfully treat a secondary dissection close to the celiac trunk's origin.

## 2. Case Presentation

**Figure 1:**
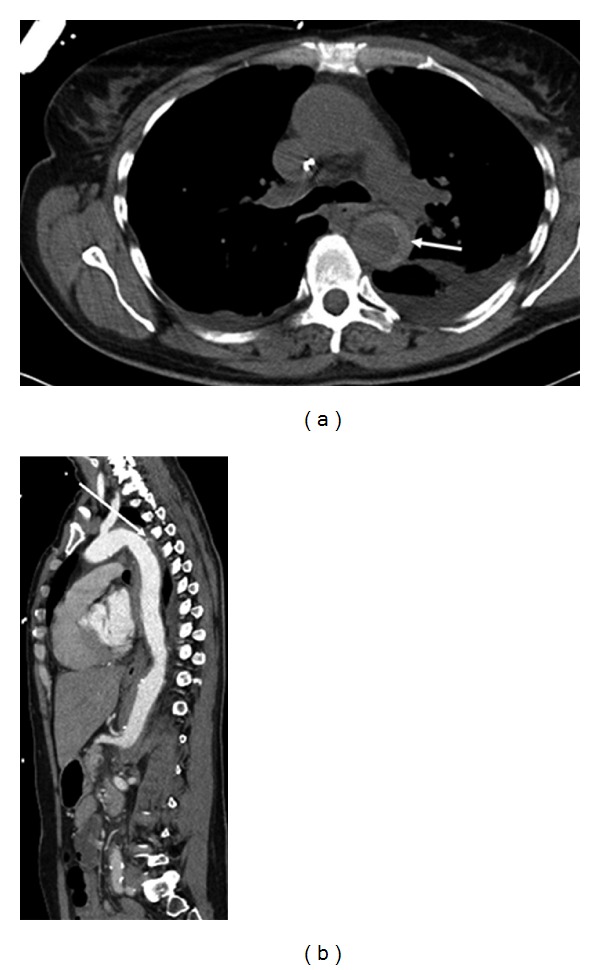
(a) Nonenhanced CT depicting intramural hematoma (arrow). (b) Sagittal reconstruction of CT-angiography (CTA) with penetrating ulcer (arrow).

A 51-year-old woman presented with acute onset of chest pain, coughing, and dyspnea. Blood pressure was elevated. She reported many years of cigarette smoking as a cardiovascular risk factor. White blood cell count was elevated with 13.22 G/l (normal: 4.4–11.3 G/l); Fibrinogen (724 mg/dL, normal: 210–400 mg/dL) and CRP were increased (237.6 mg/dL, normal: <8.0 mg/dL). Nonenhanced computed tomography (CT) (Figure 1(a)) depicted a semicircular, hyperdense vessel wall thickening, extending from right after the left subclavian artery's origin to the iliac arteries. CT-angiography (CTA) showed a small intramural contrast medium deposit in the proximal descending aorta (Figure 1(b)) as in a penetrating atherosclerotic ulcer of the aorta (PAU).

**Figure 2:**
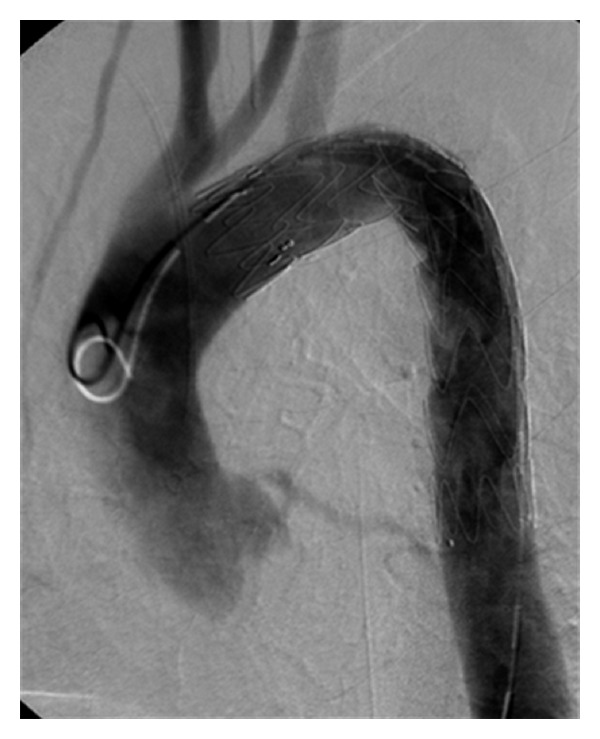
Digital subtraction angiogram (DSA) after stent graft implantation with overstenting of left subclavian artery.

A primarily conservative therapy, that is, analgesic, antihypertensive, anti-inflammatory, and antibacterial medication, did not improve the patient's symptoms: chest pain persisted while the general condition of the patient deteriorated rapidly. On day four an interdisciplinary committee decided to perform an endovascular therapy, during which a Valiant aortic stent graft (type TF 30-30-C-150, Medtronic, Fridley/MN, USA) was implanted proximal to the left subclavian artery, close to the left common carotid artery (Figure 2). Adequate cerebral perfusion was granted via the right vertebral artery which had previously been verified in MR angiography. After endovascular therapy, the patient fully recovered and was discharged 11 days after intervention with well-controlled blood pressure.

**Figure 3:**
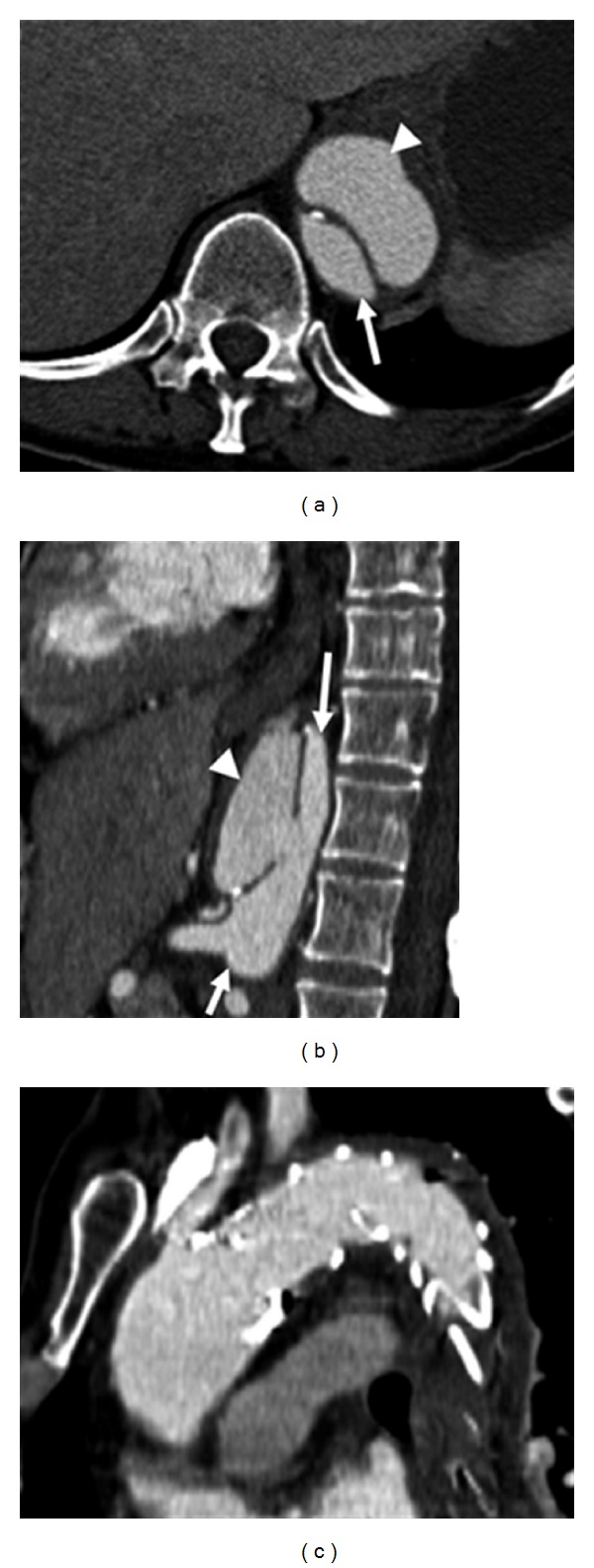
(a) CTA after three weeks after first intervention shows a newly developed dissection extending to the celiac trunk (arrow: true lumen and arrowhead: false lumen). (b) Sagittal reconstruction of the same CTA shows wide communication between the true and false lumina (arrows: true lumen and arrowhead: false lumen). (c) Coronal CTA reconstruction showing the satisfying position of the primary implanted stent graft.

**Figure 4:**
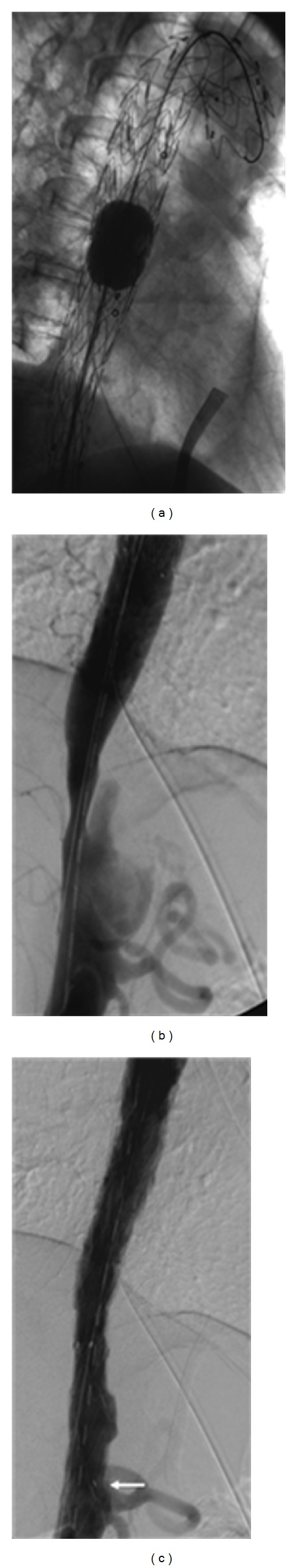
(a) and (b) DSA shows the distal extension of the stent graft using two overlapping Jotec E-vita prostheses. (c) Completion angiogram shows the distal end of the stent membrane (arrow) right at the caudal ending of the dissection, just proximal to the celiac trunk's origin.

**Figure 5:**
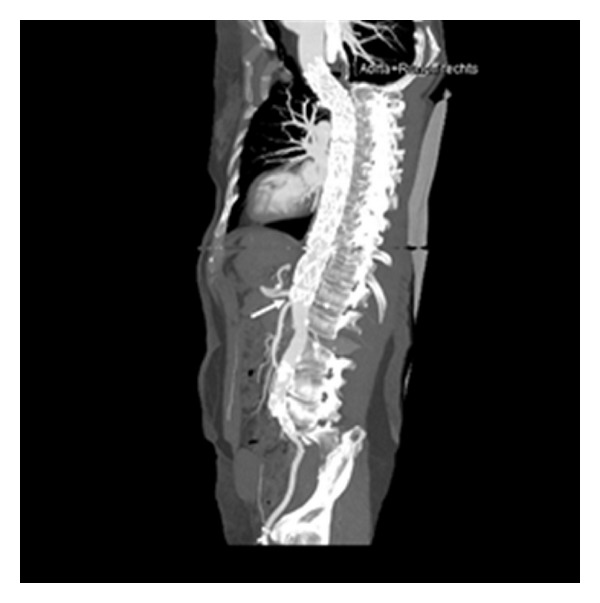
CTA after 3 years and 7 months shows that the distal end of the prosthesis membrane is located immediately proximal to the origin of the celiac trunk (centerline reconstruction, arrow), completely covering the dissection site. The celiac trunk shows physiologic contrast enhancement. No signs of recurrence are visible.

Three weeks after endovascular therapy, the patient presented with back pain and increased blood pressure. CTA depicted a newly developed circumscribed dissection of the thoracoabdominal aorta which extended almost to the celiac trunk (Figure 3(a)) with broad communication between true and false lumina (Figure 3(b)). Stent graft patency and location were optimal, and intramural hematoma was resorbed almost completely (Figure 3(c)). Despite the blood pressure had been normalized due to adapted antihypertensive medication, the whole aortic lumen diameter at the dissection site proximal to the celiac trunk increased by 1 cm (from 3.5 cm to 4.5 cm). Therefore, two months after primary intervention two overlapping Jotec E vita stent grafts (33 × 150 mm) were implanted coaxially in order to extend the primary stent graft into the abdomen. The membrane of the distal stent graft ended immediately proximal to the origin of the celiac trunk, completely covering the dissection site (Figures 4(a)–4(c)). The bare end overlapped the celiac trunk to improve fixation in the healthy vessel wall. Spinal CSF pressure was normal during and after the procedure. After coaxial distal extension of the stent graft the patient recovered rapidly. No thoracic/abdominal pain, neurological symptoms or arm claudication were reported. The patient presented free of symptoms, after 3 years and 7 months. CTA showed an excellent outcome without any signs of recurrence (Figure 5).

## 3. Discussion

Pathogenesis of aortic intramural hematoma is unclear. First described by Krukenberg in 1920, it was initially seen as hemorrhage of the aortic vasa vasorum [1]. However, recent studies in which high-resolution CT was implied indicate tearing of the aortic wall and/or penetrating ulcers with hemorrhage into the tunica media as a possible cause of at least a subgroup of intramural hematomas [2, 3]. In this case, repeated penetrations of the aortic wall are the most likely cause, with underlying elevated blood pressure and possibly preexisting vessel wall damage. The first penetration was seen as a discrete contrast agent deposit on the convexity of the aortic arch in CTA and led to an extensive intramural hematoma. The second penetration occurred after successful endovascular treatment of the first one and was so extensive that the invading blood could circulate in the tunica media which led to a progressive proximal and distal increase. Due to ongoing pain, an aortic stent graft was implanted to seal the penetration in the first place. Although stent graft placement at the point penetration is considered to be a successful treatment of intramural hematomas, our goal was to reach the healthy proximal landing zone, subsequently overstenting the left subclavian artery without any clinical consequences. The circumscribed thoracoabdominal dissection had to be treated as well due to progression. Considered therapeutic options were either open surgical reconstruction (Crawford type III) or distal coaxial endoluminal extension of the primary aortic stent graft. A reason that might make the latter option unadvisable is the expansion of the dissection close to the celiac trunk, which would have been occluded if a healthy segment of the aortic vessel wall had been chosen as landing zone. Therefore we chose to land the additional stent grafts right proximal to the celiac trunk's origin. That way the communication between true and false aortic lumen was sealed sufficiently and the dissection regressed completely.

This example proves the efficiency of placing the distal membrane of the aortic stent graft within the affected segment in individual cases. However, in general sufficient contact between the covered segment and the healthy vessel wall should be achieved [4].
